# Prognostic value of serum/plasma neurofilament light chain for COVID‐19‐associated mortality

**DOI:** 10.1002/acn3.51542

**Published:** 2022-03-21

**Authors:** Ruturaj R. Masvekar, Peter Kosa, Kimberly Jin, Kerry Dobbs, Michael A. Stack, Riccardo Castagnoli, Virginia Quaresima, Helen C. Su, Luisa Imberti, Luigi D. Notarangelo, Bibiana Bielekova

**Affiliations:** ^1^ National Institute of Allergy and Infectious Diseases (NIAID), National Institutes of Health (NIH) Bethesda Maryland USA; ^2^ CREA Laboratory (AIL Center for Hemato‐Oncologic Research), Diagnostic Department ASST Spedali Civili di Brescia Brescia Italy

## Abstract

**Objective:**

Given the continued spread of coronavirus 2, the early predictors of coronavirus disease 19 (COVID‐19) associated mortality might improve patients' outcomes. Increased levels of circulating neurofilament light chain (NfL), a biomarker of neuronal injury, have been observed in severe COVID‐19 patients. We investigated whether NfL provides non‐redundant clinical value to previously identified predictors of COVID‐19 mortality.

**Methods:**

We measured serum or plasma NfL concentrations in a blinded fashion in 3 cohorts totaling 338 COVID‐19 patients.

**Results:**

In cohort 1, we found significantly elevated NfL levels only in critically ill COVID‐19 patients. Longitudinal cohort 2 data showed that NfL is elevated late in the course of the disease, following the two other prognostic markers of COVID‐19: decrease in absolute lymphocyte count (ALC) and increase in lactate dehydrogenase (LDH). Significant correlations between ALC and LDH abnormalities and subsequent rise of NfL implicate that the multi‐organ failure is the most likely cause of neuronal injury in severe COVID‐19 patients. The addition of NfL to age and gender in cohort 1 significantly improved the accuracy of mortality prediction and these improvements were validated in cohorts 2 and 3.

**Interpretation:**

A substantial increase in serum/plasma NfL reproducibly enhanced COVID‐19 mortality prediction. Combined with other prognostic markers, such as ALC and LDH that are routinely measured in ICU patients, NfL measurements might be useful to identify the patients at a high risk of COVID‐19‐associated mortality, who might still benefit from escalated care.

## Introduction

Since early 2020, the coronavirus disease 19 (COVID‐19) pandemic has exhausted medical systems worldwide. Even after the development of safe and effective vaccines, severe acute respiratory syndrome coronavirus 2 (SARS‐CoV‐2) continues to spread.^1^ A reliable early predictor of COVID‐19 associated mortality would help prioritize use of medical resources and maximize patient survival.

Neurofilaments are essential cytoskeleton proteins of the central and peripheral axons exclusive to the nervous system. Compared to neurofilament heavy and medium chains (NfH and NfM, 200 and 150 kDa), neurofilament light chain (NfL, 68 kDa) has a lower molecular weight and easily diffuses from parenchyma to cerebrospinal fluid (CSF) and blood.^2^, ^3^, ^4^ Recent developments of ultrasensitive assays, such as single molecule array (SIMOA), allow reproducible measurement of low NfL concentrations in serum or plasma.^5^, ^6^ Consequently, blood NfL became a key noninvasive biomarker of acute neuronal injury in diverse neuropathological conditions,^7^ including sepsis‐associated encephalopathy.^8^

Although previous studies have demonstrated an association between COVID‐19 morbidity and central nervous system (CNS) damage,^9^, ^10^, ^11^, ^12^, ^13^ several questions still remain unanswered: (1) Does a single measurement of NfL provide meaningful prognostic information at individual patient level?; (2) Is there a relationship between NfL and previously described COVID‐19‐associated mortality biomarkers^14^ of prognostic value, such as absolute lymphocyte count (ALC), C‐reactive protein (CRP), and lactate dehydrogenase (LDH)?; and (3) Does NfL improve COVID‐19 mortality prediction by demographic markers such as age and gender?

## Materials and Methods

### Research subjects and cohorts

Serum or plasma samples from COVID‐19 patients admitted at ASST Spedali Civili (Brescia, Italy) were obtained through the Laboratory of Clinical Immunology and Microbiology (LCIM), National Institute of Allergy and Infectious Diseases (NIAID), under Institutional Review Board (IRB)‐approved protocols (Comitato Etico Provinciale: NP 4000 – Studio CORONAlab and NP 4408 – Studio CORONAlab and ClinicalTrials.gov: NCT04582903). Blood samples from all patients were taken between 6:00 and 11:00 AM; samples were collected in S‐Monovette® serum and S‐Monovette® lithium heparin (Catalog # 04.1934.001 and 04.1936; Sarstedt, Numbrecht, Germany) tubes for isolation of serum and plasma, respectively. Tubes were centrifuged at 2000*g* for 10 min at 20°C, samples were collected, aliquoted, and stored at −80°C; frozen samples were shipped on dry ice.

SARS‐CoV‐2 infection was confirmed using the nasopharyngeal swab – polymerase chain reaction test. COVID‐19 disease severity was determined as per Diagnosis and Treatment Protocol for Novel Coronavirus Pneumonia guidelines, released by the National Health Commission & State Administration of Traditional Chinese Medicine.^15^

Serum and plasma samples from healthy controls (HC) and multiple sclerosis (MS) subjects were collected at Neuroimmunological Diseases Section (NDS), NIAID after informed consent under IRB approved protocol (ClinicalTrials.gov: NCT00794352). The NfL levels measured in HC and MS subgroups were previously reported^16^ and are used in the current study only as a positive control of neuronal injury; the measurements of other COVID‐19 prognostic biomarkers in these control samples were not reported previously.

**Figure 1:**
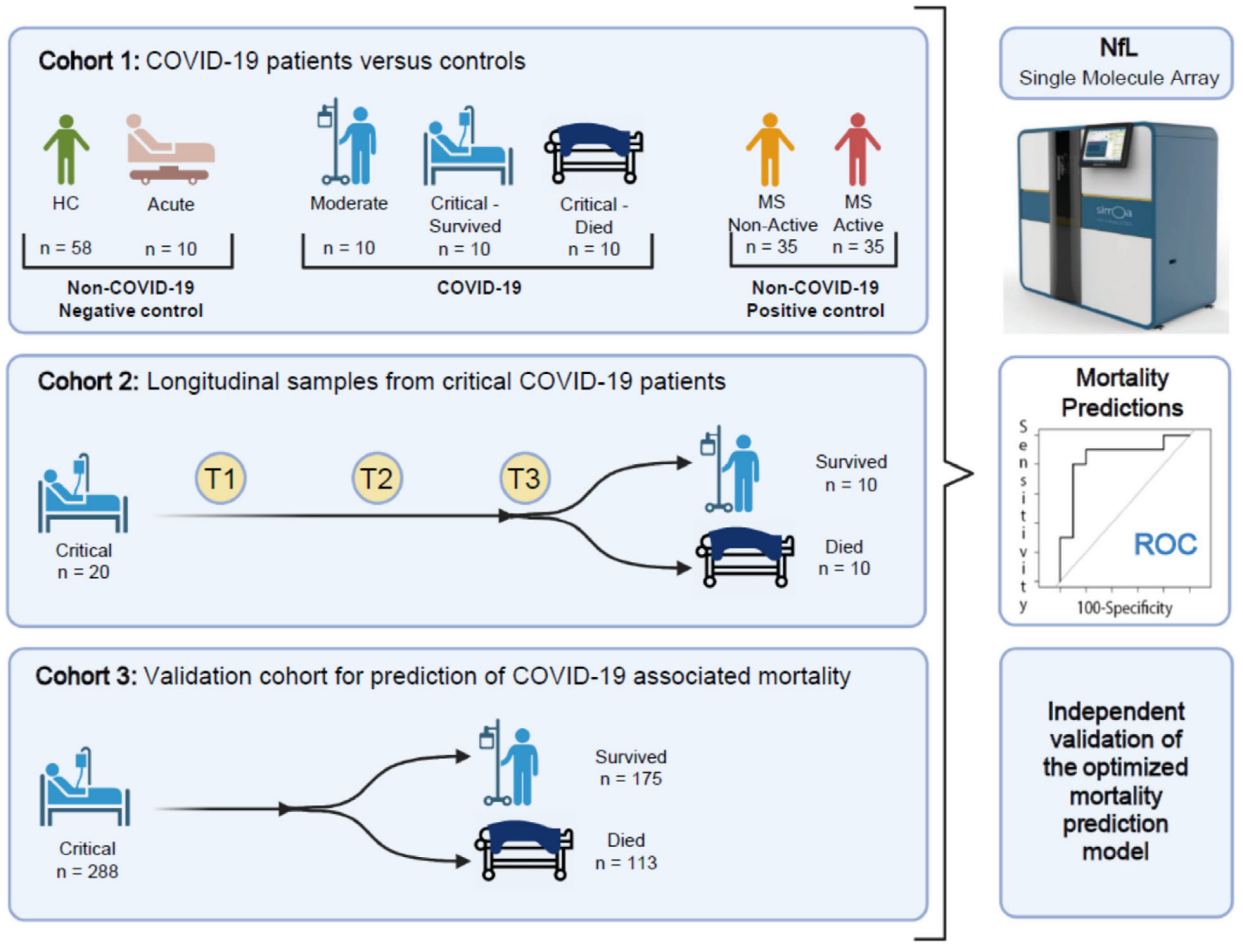
Patient selection, objectives, and experiment outlines of 3 independent cohorts. Cohort 1 aims to analyze NfL cross‐sectionally across disease diagnosis and severity categories. In cohort 2, the objective was to analyze NfL levels in critically ill COVID‐19 patients, longitudinally at three different timepoints (T1, T2, and T3: collected averagely at 5‐ to 10‐day interval, within 30 days of hospitalization). Observed additional prognostic value of NfL with traditional demographic factors (age and gender) from cohorts 1 and 2, was independently validated in cohort 3. NfL, neurofilament light chain; COVID‐19, coronavirus disease 19. This figure was generated using BioRender.com software. [Colour figure can be viewed at wileyonlinelibrary.com]

Three hundred seventy‐eight serum or plasma samples were collected from 338 COVID‐19 patients grouped into three independent cohorts (Fig. 1, Table 1, and Data S1). In cohort 1, 30 cross‐sectional serum samples were collected from COVID‐19 patients with three levels of disease severity. In cohort 2, 60 longitudinal plasma samples were collected from 20 critically ill COVID‐19 patients (T1, T2, and T3: collected averagely at 5‐ to 10‐day intervals, within 30 days of hospitalization). Cohort 3 consisted of 288 cross‐sectional plasma samples collected from critically ill COVID‐19 patients where a large proportion of the subjects eventually died (39.2%).

**Table 1:** Demographic details of the three cohorts.

			Non‐COVID‐19				COVID‐19		
			Negative Control		Positive Control (MS)		Moderate	Critical	
			HC	Acute	Non‐active	Active		Survived	Died
Cohort 1 (*N* = 168) (serum)	*N*		58	10	35	35	10	10	10
	Age (years)	Mean (SD)	41.1 (13.7)	43.6 (20.8)	52.8* (11.3)	37.6 (10.8)	51.8 (13.9)	56.1* (11.4)	69.4* (11.7)
	Gender (female)	*n* (%)	28 (48.3)	2 (20.0)	16 (45.7)	22 (62.9)	3 (30.0)	2 (20.0)	1* (10.0)
	Comorbidities (yes)	*n* (%)	–	7 (70.0)	–	–	8 (80.0)	7 (70.0)	9 (90.0)
Cohort 2 (*N* = 38) (plasma)	*N*		18	–	–	–	–	10	10
	Age (years)	Mean (SD)	44.1 (10.5)	–	–	–	–	64.1* (9.9)	67.0* (6.9)
	Gender (female)	*n* (%)	9 (50.0)	–	–	–	–	5 (50.0)	0*^,#^ (0.0)
	Comorbidities (yes)	*n* (%)	–	–	–	–	–	6 (60.0)	10^#^ (100.0)
Cohort 3 (*N* = 288) (plasma)	*N*		–	–	–	–	–	175	113
	Age (years)	Mean (SD)	–	–	–	–	–	73.8 (10.7)	77.3^#^ (10.4)
	Gender (female)	*n* (%)	–	–	–	–	–	40 (22.8)	35 (30.9)
	Comorbidities (yes)	*n* (%)	–	–	–	–	–	123 (70.3)	87 (76.9)

Age (unpaired *t*‐test), gender, and comorbidities (Chi‐square test) were compared across disease diagnosis/severity subgroups. COVID‐19, coronavirus disease 19; HC, healthy controls; MS, multiple sclerosis.

*p < 0.05 versus HC and ^#^ *p* < 0.05 versus COVID‐19, critical – survived.

### NfL single molecular array (Simoa™) assay

Frozen serum and plasma samples were thawed on ice and were used immediately; repeated freezing and thawing of the samples was avoided. NfL concentrations in samples were measured using the Simoa™ assay (Catalog # 103186; Quanterix, Billerica, MA, USA). Samples were diluted 1:4 and randomly distributed on 96‐well plates. Quality control (QC) samples provided with the kit had concentrations within the pre‐defined range and the coefficient of variance (CV) across the plates was <10%: (1) cohort 1: 1 plate, no CV; (2) cohort 2: 3 plates, control 1 = 8.9% and control 2 = 3.7%; (3) cohort 3: 5 plates, control 1 = 8.6% and control 2 = 7.6%. All samples were analyzed blindly under alpha‐numeric codes. The diagnostic codes were broken only after QC‐verified NfL concentrations were reported to the database manager.

To determine the effect of sample age on NfL degradation and assay performance, within each cohort and disease diagnosis/severity subgroups, we analyzed the correlations between sample age (date of sample analysis – date of sample collection, in days; Data S1) and NfL concentrations (pg/mL) using linear regression models. We did not observe statistically significant (*p* < 0.05) correlations (data not shown).

### Adjustment for the effect of healthy aging

As serum/plasma NfL levels increase with physiological aging,^17^ the measured NfL concentrations were adjusted for the effect of healthy aging as described previously.^16^ Following age versus serum‐ or plasma‐NfL equations from HC cohorts were used: ln(serum NfL) = 0.0177 × Age + 0.9696 and ln(plasma NfL) = 0.0158 × Age + 1.247. The age‐adjusted NfL concentrations represent residuals from the above‐stated linear regression models.

### Statistical analyses

NfL levels were compared across disease diagnosis and severity subgroups using either Kruskal–Wallis ANOVA or Welch's *t*‐test. Correlations between NfL and systemic markers of COVID‐19 morbidity were evaluated using Spearman analysis and linear regression model.

Prediction models of COVID‐19‐associated mortality were developed in R Studio Version 1.1.463 (R version 4.0.2) using logistic regression (*glm* function of the “stat” package).^18^ Optimal cutoff for the predictive models was calculated using the *optimalCutoff* function of the “InformationValue” package (https://cran.r‐project.org/web/packages/InformationValue/index.html). The area under the receiver operating characteristic curve (AUROC) was calculated using the *roc* function of the “pROC” package.^19^ AUROC measures model's ability to discriminate between positive cases versus negative cases, AUROC >0.7 is considered a good performing model.

## Results

### NfL levels increase with COVID‐19 severity and mortality

Although increased blood NfL levels have been reported in patients with severe COVID‐19,^9^, ^10^, ^11^, ^12^, ^13^ previous studies had insufficient numbers of subjects who died from the disease to assess whether NfL can predict COVID‐19 mortality.

To fill this knowledge gap, we measured NfL levels in 30 COVID‐19 patients with three levels of severity: (1) moderate severity (*n* = 10); (2) critical condition but survived (*n* = 10); and (3) critical condition but died (*n* = 10). Positive and negative control subgroups consisted of (1) patients with acute COVID‐19‐like symptoms admitted in critical health conditions who tested negative for SARS‐CoV‐2 infection (*n* = 10); (2) HC (*n* = 58); (3) MS patients with acute focal CNS inflammation measured as contrast‐enhancing lesions on brain MRI (active MS, *n* = 35); and (4) MS patients without evidence of acute focal CNS inflammation (non‐active, *n* = 35).

**Figure 2:**
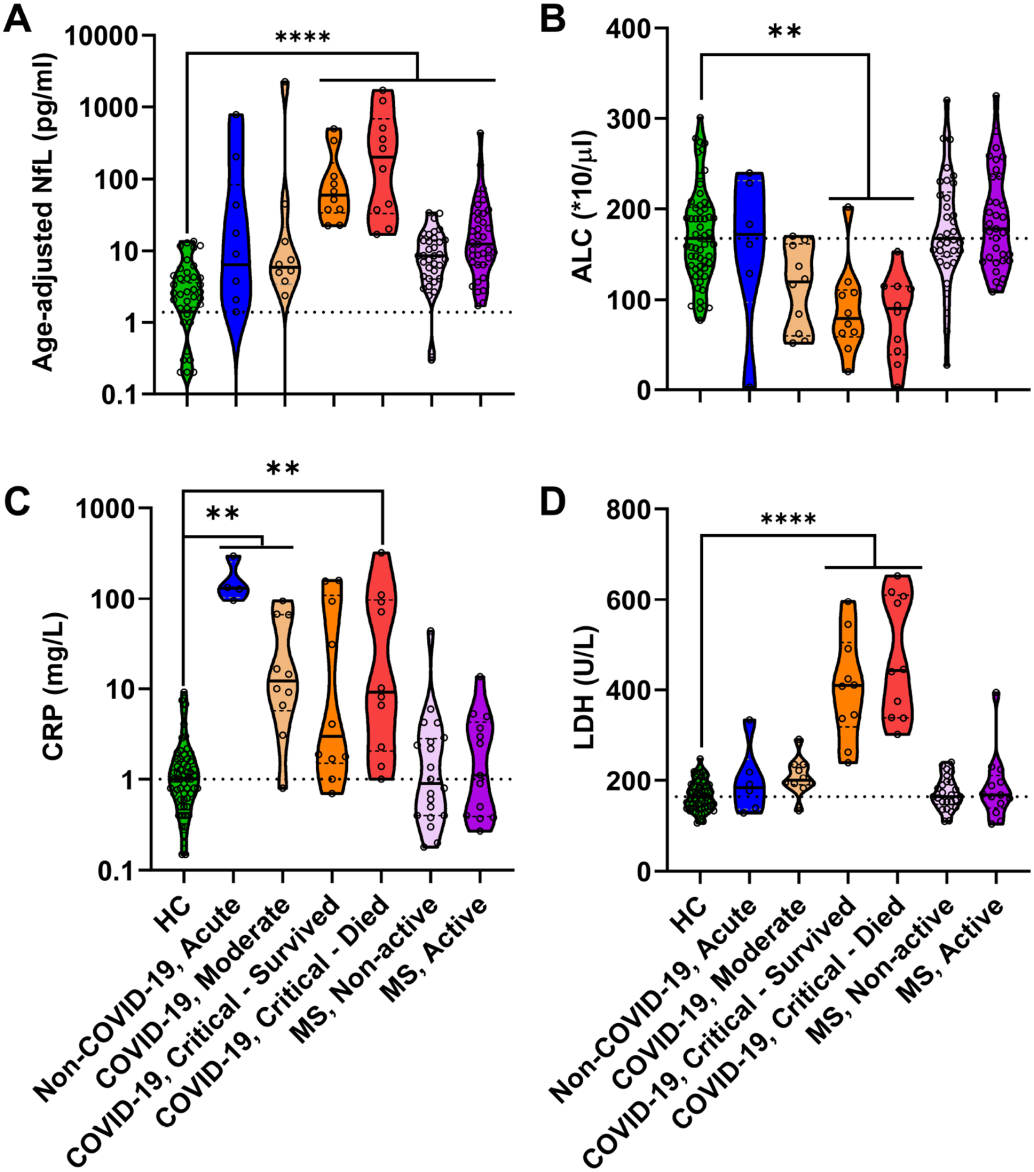
In cohort 1, serum (A) NfL, (B) ALC, (C) CRP, and (D) LDH levels were compared across HC versus COVID‐19 disease severity and multiple sclerosis disease activity subgroups using Kruskal–Wallis ANOVA; **p < 0.005 and ****p < 0.0001. The dotted line on each plot indicates the median of HC. NfL, neurofilament light chain; ALC, absolute lymphocyte count; CRP, C‐reactive protein; LDH, lactate dehydrogenase; COVID‐19, coronavirus disease 19; HC, healthy controls. [Colour figure can be viewed at wileyonlinelibrary.com]

After diagnostic codes were unblinded, we found elevated levels of NfL in COVID‐19 patients compared to HC (Fig. 2A). NfL levels in COVID‐19 patients increased with disease severity, but only cohorts of critically ill COVID‐19 and MS patients reached statistical significance compared to HC.

Next, we compared cohort differences in other blood biomarkers of COVID‐19 morbidity: ALC, CRP, and LDH (Fig. 2B–D). Like NfL, decreased ALC and increased LDH correlated with COVID‐19 severity; statistically significant differences in ALC and LDH were observed only in critically ill COVID‐19 patients compared to HC. Interestingly, although non‐COVID‐19 acute respiratory illness control had levels of COVID‐19 prognostic biomarkers (i.e., NfL, ALC, and LDH) comparable to HC, they had the highest levels of the prototypical acute phase reactant, CRP.

We conclude that NfL, LDH, and ALC abnormalities increase with COVID‐19 severity, are associated with COVID‐19 mortality, and can differentiate COVID‐19 from other acute respiratory conditions that lead to ICU admission.

### In COVID‐19 patients NfL rises later during hospitalization, trailing transient abnormalities in ALC and LDH by 5–20 days

The earlier a biomarker can identify patients at risk for COVID‐19 mortality, the greater its clinical value. Because none of the previous studies addressed the dynamics of NfL rise in COVID‐19 and compared it to the dynamics of other prognostic biomarkers, we addressed this knowledge gap in the longitudinal cohort 2.

**Figure 3:**
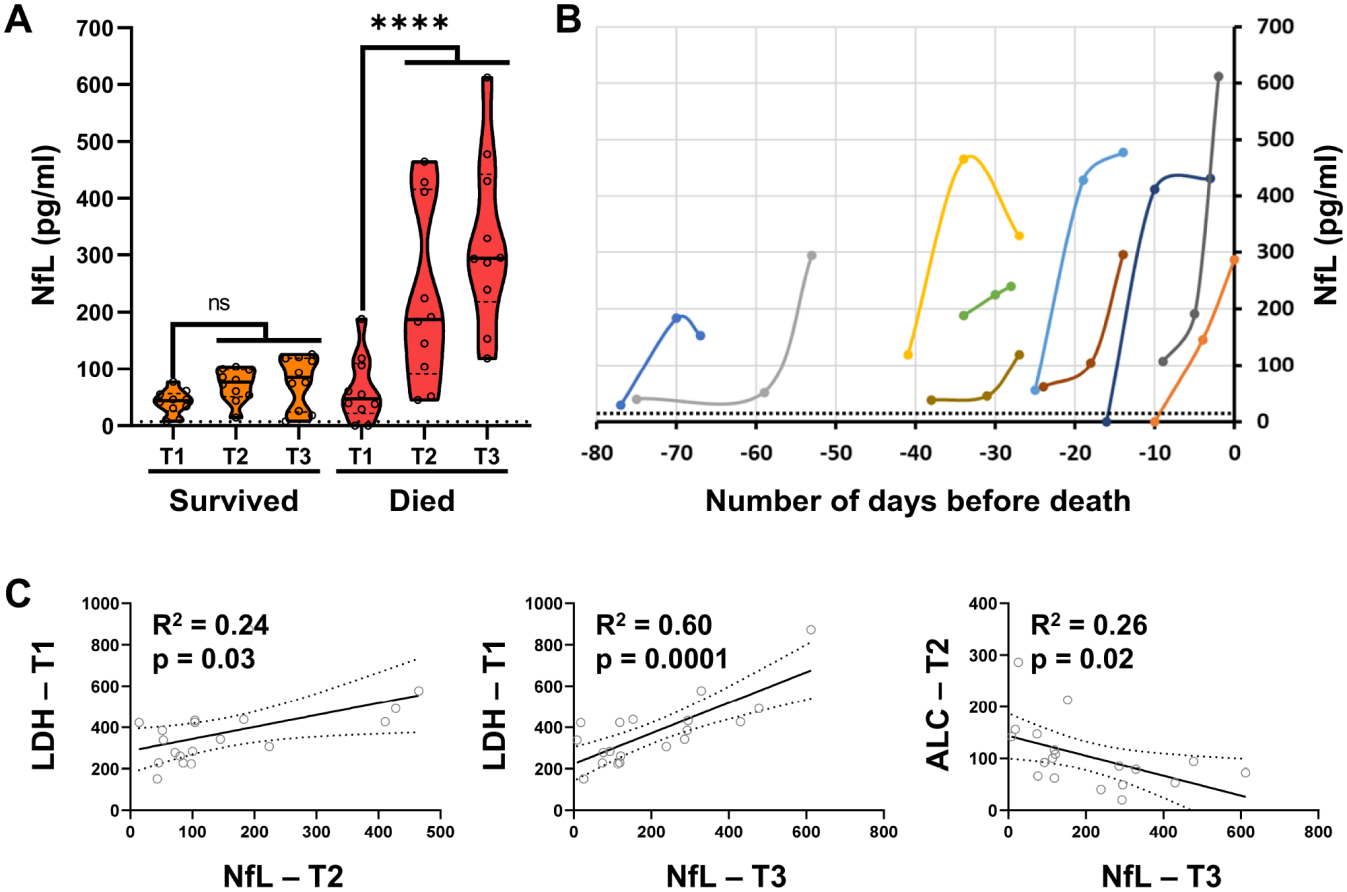
In cohort 2, (A) plasma NfL levels at 3 different time points (T1, T2, and T3: collected on average at 5‐to‐10‐day intervals, within 30 days of hospitalization) in critically ill COVID‐19 patients were compared (survived vs. died) using Kruskal–Wallis ANOVA; ****p < 0.0001. The dotted line indicates the median of the HC. (B) Longitudinal plasma NfL levels in critical COVID‐19 patients who died, plotted with respect to the number of days before death. Each line represents data from an individual patient. The dotted line represents upper limit in HC (i.e., mean + 3 × SD = 20 pg/mL). (C) Correlations between systemic biomarkers' measurements at earlier time points (T1 and 2) and NfL measurements at later time points (T2 and T3) were assessed using linear regression analysis. *R* ^2^ and *p*‐value are represented on respective correlation plots. The dotted line indicates a 95% confidence interval. NfL, neurofilament light chain; COVID‐19, coronavirus disease 19; HC, healthy controls. [Colour figure can be viewed at wileyonlinelibrary.com]

We measured NfL in 60 samples collected from 20 critically ill COVID‐19 patients within 30 days of hospitalization, at three timepoints (T1, T2, and T3) taken at approximately 5‐ to 10‐day intervals. We observed statistically significant, progressive increases (T1 vs. T2 and T3) in NfL levels only in patients who later died (Fig. 3A and Appendix S1).

**Figure 4:**
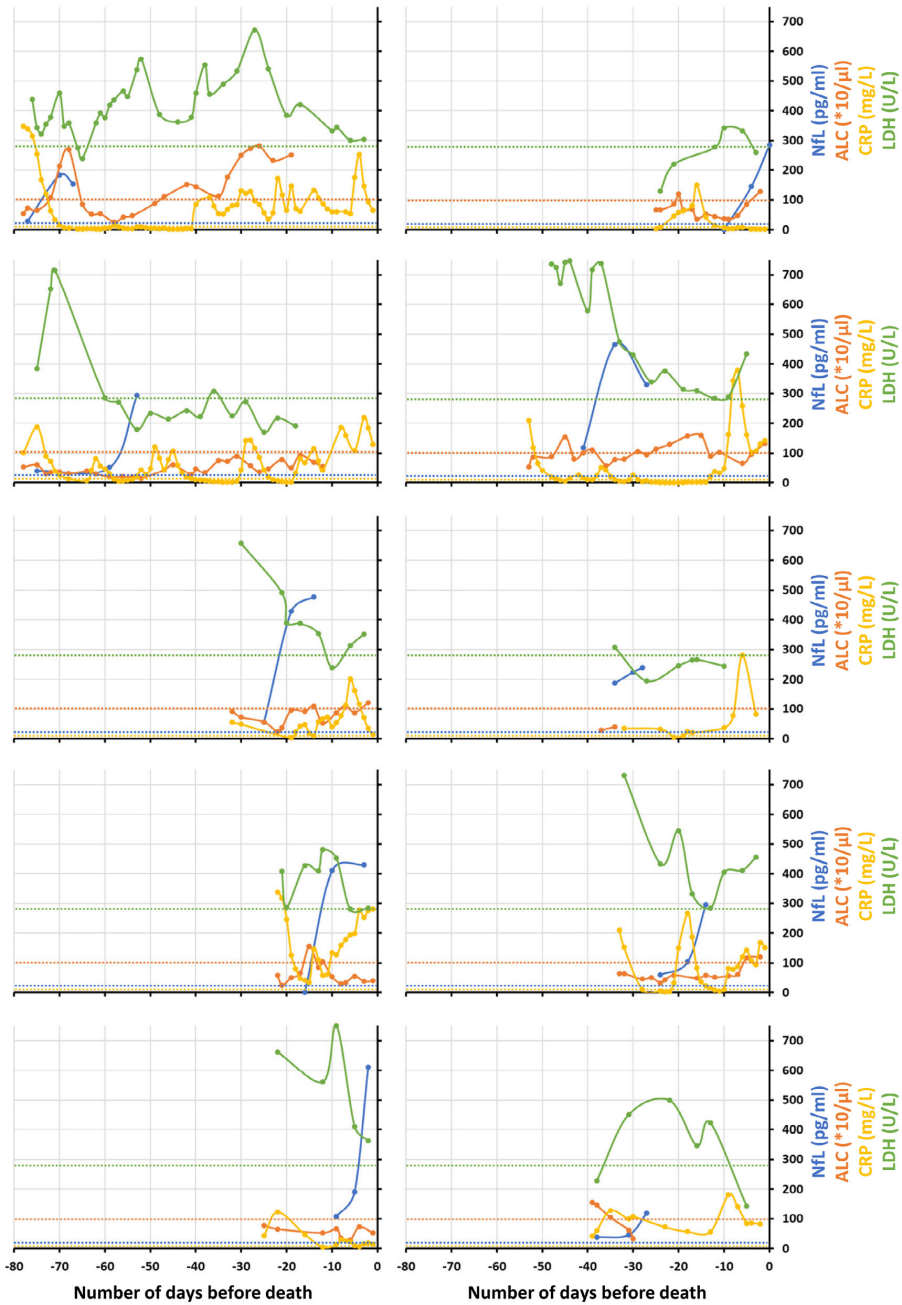
In cohort 2, longitudinal plasma NfL (blue), ALC (orange), CRP (yellow), and LDH (green) levels in critically ill COVID‐19 patients those who died, plotted with respect to the number of days before death. Each plot represents individual patient data. The respective color dotted lines represent upper (for NfL, CRP, and LDH) or lower (for ALC) limit for HC for the respective biomarker (NfL: 20 pg/mL, ALC: 100 × 10/μL, CRP: 5 mg/L and LDH: 280 U/L). NfL, neurofilament light chain; ALC, absolute lymphocyte count; CRP, C‐reactive protein; LDH, lactate dehydrogenase; COVID‐19, coronavirus disease 19; HC, healthy controls. [Colour figure can be viewed at wileyonlinelibrary.com]

When plotting measurements against the number of days before death, we observed a progressive increase in NfL approaching death, while no such increases occurred in subjects who eventually survived (Figs. 3B, 4 and Fig. S1). Consistent with prior reports that NfL levels remain elevated for weeks (up to 3 months) following acute CNS injury,^20^ increased NfL in COVID‐19 patients did not return to normal within the observation period. In contrast, ALC, LDH, and CRP demonstrated large day‐to‐day fluctuations (Fig. 4) and were also frequently elevated in surviving patients (Fig. S1 and Appendix S1).

To assess if transient abnormalities in LDH, CRP, and ALC levels precede increases in NfL, we investigated correlations between these systemic markers measured at initial timepoints (T1 and T2), with NfL measured later (i.e., T1 vs. T2, T1 vs. T3, and T2 vs. T3). Only three of these comparisons reached statistical significance (Fig. 3C), with the strongest relationship observed between LDH measured at first time point (T1) and NfL measured at last time point (T3), which explains almost 60% of variance (*R* ^2^ = 0.598, *p* = 0.0001). Consistent with the lack of association of CRP measurements with COVID‐19 severity, CRP elevations did not predict subsequent rise in NfL.

We conclude that critically ill COVID‐19 patients experience earlier abnormalities in ALC and LDH measurements, which are strongly associated with later elevation in NfL levels.

### NfL measured later during hospitalization enhances mortality prediction of age‐ and gender‐based classifier

As all the above‐described observations supported the clinical value of NfL to predict COVID‐19 mortality, we sought to quantify this predictive value on an individual patient level and compare it to demographic prognostic markers such as age, gender, and comorbidities.

**Figure 5:**
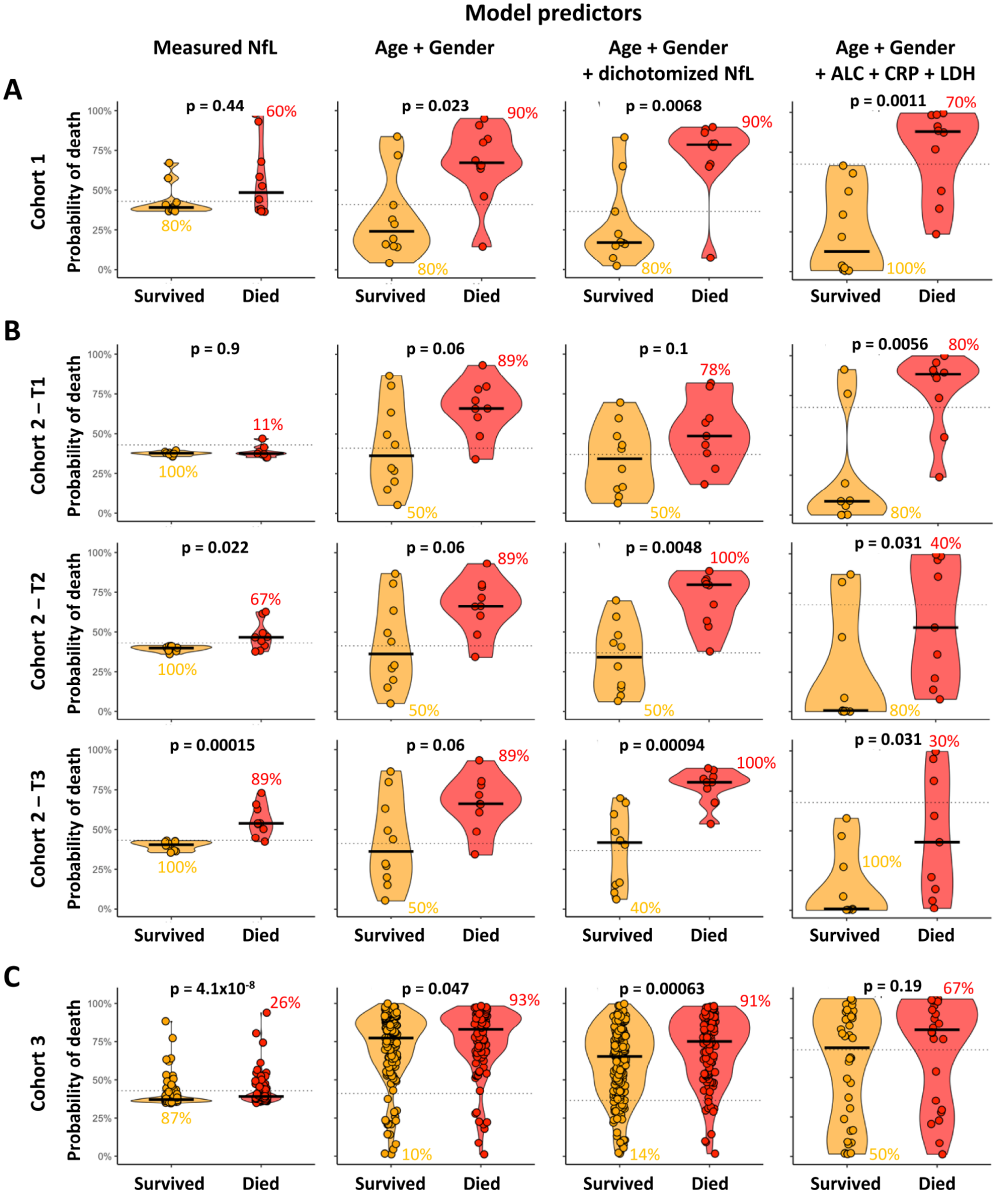
Comparisons of 4 predictive models of COVID‐19 associated mortality: continuous NfL measurement, Age plus Gender, Age plus Gender plus dichotomized NfL, and Age plus Gender plus ALC plus LDH plus CRP in 3 independent cohorts; (A) cohort 1, (B) cohort 2, and (C) cohort 3. The dotted line on each plot represents the optimal cut‐off for respective model predictor. The numerical values beside respective subgroups (survived or died) on each plot represents the percentage of correctly classified patients. In validation cohort 2, one subject from Died subgroup was repeated from training cohort 1, so that subject was excluded during validation. In validation cohort 3, all three laboratory (ALC, LDH, and CRP) values were available only for 62 subjects (survived = 38 and died = 24), so only these subjects were included in validation (cohort 3: column 4). NfL, neurofilament light chain; ALC, absolute lymphocyte count; CRP, C‐reactive protein; LDH, lactate dehydrogenase; COVID‐19, coronavirus disease 19. [Colour figure can be viewed at wileyonlinelibrary.com]

In cohort 1, used as a training cohort, we predicted COVID‐19 mortality using measured NfL as a continuous variable (Fig. 5A, left panel). Single, cross‐sectional NfL measurements could not reliably predict death, reaching an area under receive operator characteristic curve (AUROC) of only 0.61 with a 95% confidence interval ([CI]: 0.33–0.89) crossing the value of random guessing (i.e., AUROC 0.5). The optimal cut‐off from NfL to predict mortality from cohort 1 ROC curve was 124 pg/mL.

As shown in Table 1, cohorts 1 and 2 were not matched for demographic predictors of COVID‐19 mortality: in both cohorts, patients who survived were generally younger, with a higher proportion of females and a lower proportion of subjects with comorbidities. Therefore, it should not be surprising that NfL measurements alone, ignoring these important demographic variables, had low predictive power. Instead, we built a prognostic classifier that integrated NfL (dichotomized based on optimal cut‐off 124 pg/mL) with age and gender and compared it to the model(s) without NfL. We also tested a more complex classifier consisting of dichotomized NfL, age, gender, and comorbidities, but observed weaker independent validation of this model compared to a model without comorbidities (Fig. S2). For the sake of space and clarity, we will present data only on the strongest model.

Adding dichotomized NfL enhanced the predictive value of age and gender in cohort 1 from AUROC 0.80 (CI: 0.58–1.00) to 0.85 (0.66–1.00) and *p*‐value from 0.023 to 0.0068 (Fig. 5A and Fig. S3A).

Next, we sought to assess the performance of the leading mortality predictor in cohort 2, which did not contribute to model generation (Fig. 5B and Fig. S3B). The addition of dichotomized NfL to the age and gender at first longitudinal timepoint (T1) did not improve the predictive value of the model, consistent with the observation that at the early timepoint the NfL values were indistinguishable between patients who survived and those who died. In contrast, NfL significantly improved the predictive power of the combined classifier at later timepoints (T2 and T3; T2: AUROC from 0.76 (CI: 0.53–1.00) to 0.89 (CI: 0.74–1.00) and *p*‐value from 0.06 to 0.0048; T3: AUROC from 0.76 (0.53–1.00) to 0.96 (0.87–1.00) and *p*‐value from 0.06 to 0.00094).

We conclude that NfL measurement provides additive COVID‐19 mortality predictive value to the traditional demographic prognostic factors, provided that NfL is measured in critically ill patients later in the disease.

Finally, we were able to assess the non‐redundant prognostic value of NfL in a unique large cohort of patients with high COVID‐19 mortality risk (i.e., elderly patients with high proportion of males with comorbidities; Fig. 5C). As expected, out of these 288 critically ill COVID‐19 patients, a large proportion (*n* = 113; 39.2%) eventually died.

Although surviving and dying cohorts were matched for age, gender, and comorbidities as univariate predictors (Table 1), the combined age plus gender model correctly predicted marginally higher mortality in the cohort of subjects who eventually died (10% vs. 93%; *p* = 0.047). NfL levels differentiated survivors from non‐survivors with much stronger statistical significance (*p* = 4.1e‐08). Adding dichotomized NfL to demographic data improved the accuracy of mortality prediction compared to demographic data alone. Specifically, the AUROC increased from 0.57 (CI: 0.50–0.64) to 0.62 (CI: 0.55–0.69) and *p*‐value improved from 0.047 to 0.00063 (Fig. 5C and Fig. S3C). Nevertheless, the sensitivity (71.4%) and specificity (40.7%) of this predictor remained weak in this unique cohort.

The LDH, CRP, and lymphopenia were previously associated with COVID‐19 mortality, especially in Chinese patients where a tree‐based classifier (XGBoost) that included all three of these biomarkers achieved greater than 90% accuracy in predicting death in the independent validation cohort.^14^ Unfortunately, this model failed to validate in a cohort of Dutch (Caucasian) patients.^21^ As we did observe in univariate analyses prognostic value of LDH and ALC in our Caucasian (i.e., Italian) cohort, we construed a model that included age, gender, and these three biomarkers using modeling strategy analogous to our best validated NfL mortality predictor (Fig. 5: column 4 and Fig. S3: column 3). This model outperformed the winning NfL model in the training cohort (cohort 1), achieving AUROC of 0.91 (CI: 0.79–1.00) and *p* = 0.0011. However, consistent with our univariate observation, this last classifier outperformed the winning NfL model only in the earliest timepoint (T1) of independent longitudinal cohort 2 (T1: AUROC = 0.88 [CI: 0.69–1.00] and *p* = 0.0056). Its performance was inferior to the winning NfL model in cohort 2 for later timepoints (T2: AUROC = 0.80 [CI: 0.59–1.00] and *p* = 0.031; T3: AUROC = 0.80 [0.59–1.00] and *p* = 0.031). Finally, the model containing LDH, ALC, and CRP completely failed to validate in cohort 3 (AUROC = 0.60 [CI: 0.45–0.75] and *p* = 0.19 [Fig. 5 and Fig. S3]), although one must note that we did not have these laboratory values for all subjects in cohort 3.

## Discussion

An increase in serum or CSF NfL has been previously associated with increased ICU mortality due to sepsis‐associated encephalopathy.^8^ This study expands these data to COVID‐19 ICU admissions: First, we validated reports linking high serum/plasma NfL levels to COVID‐19 severity.^9^, ^10^, ^11^, ^12^, ^13^, ^22^, ^23^ Our longitudinal measurements demonstrated that rise in NfL generally occurs during hospitalizations of critically ill patients and trails other transient laboratory abnormalities such as decreased ALC and increased LDH by 5–20 days. The degree of LDH increase is a strong determinant of the subsequent magnitude of NfL rise, suggesting that COVID‐19‐associated CNS injury is secondary to damage of other critical organs, such as liver, kidneys, and lungs. This conclusion aligns with pathology studies ruling out strong primary infiltration of CNS tissue by the SARS‐CoV‐2 or by the immune system; those studies instead attribute COVID‐19‐associated CNS damage to processes such as hypoxia or intravascular coagulation.^24^

Compared to previous studies of NfL in COVID‐19,^9^, ^10^, ^11^, ^12^, ^13^, ^22^, ^23^ we studied a cohort of patients in which a high proportion eventually died (133/338 = 39.3%). This allowed us to unequivocally link high serum/plasma NfL levels with COVID‐19 mortality, something that remained ambiguous in the previous studies.

We constructed a model that combined demographic predictors of COVID‐19 mortality with NfL measurement and validated its greater predictive accuracy. Nevertheless, the accuracy of this classifier varied between the cohorts, depending on the timing of NfL measurement (i.e., later measurements enhanced predictive power) and underlying premorbid risk. Indeed, comparing model performance among our three cohorts, it appeared that NfL has greater predictive value in younger (cohorts 1 and 2) versus older (cohort 3) subjects. This is perhaps not surprising as younger patients with fewer comorbidities have a higher likelihood of withstanding multi‐organ failure and therefore CNS injury may become a key determinant of their survival. In contrast, elderly subjects with high premorbid risk and greater vulnerability of CNS tissue to sepsis‐associated injury rapidly succumb to multi‐organ failure before CNS injury manifests by high NfL concentrations.^25^

Although speculative at the moment, integrating all our observations, we recommend that NfL should be measured longitudinally and integrated with existing prognostic markers to optimize care. For example, a screening NfL measurement at the beginning of hospitalization, expected to be normal in most patients, might identify a few subjects with either neurological comorbidity or with advanced stage of COVID‐19 who require care focused on preventing further CNS injury. After an initial negative NfL test, critically ill COVID‐19 patients might be best monitored by standard laboratory tests such as LDH and ALC. Identified spikes should prompt more aggressive management that includes longitudinal NfL monitoring approximately every 5 days. Any increase in NfL should be considered a poor prognostic indicator necessitating escalation therapies, including neuroprotective strategies. Stabilization of NfL levels indicates that escalation therapy worked, while further increases signify continuous neuro‐axonal injury that must be stopped to limit mortality.

While the COVID‐19 pandemic demonstrated the prognostic value of NfL in critically ill patients with SARS‐CoV‐2 infection, noninvasive, ultrasensitive measurement of NfL could be used to monitor neuronal injury in all comatose, or heavily sedated critically ill patients regardless of SARS‐CoV‐2 infection status. Ultra‐sensitive assays will hopefully become broadly adopted by clinical laboratories and might include in the future other CNS‐derived analytes for enhanced accuracy of noninvasive monitoring of CNS tissue.

## Conflicts of Interest

The authors declare no conflicts of interest.

## Supporting information


**Figure S1.** In cohort 2, longitudinal plasma NfL (blue), ALC (orange), CRP (yellow), and LDH (green) levels in critically ill COVID‐19 patients those who survived, plotted with respect to the number of days before discharge.

**Figure S2.** Comparisons of two predictive models of COVID‐19 associated mortality: age plus gender plus dichotomized NfL and age plus gender plus dichotomized NfL plus dichotomized comorbidities in 3 independent cohorts; (A) cohort 1, (B) cohort 2, and (C) cohort 3.

**Figure S3.** Comparisons of 3 predictive models of COVID‐19 associated mortality: age plus Gender, Age plus Gender plus dichotomized NfL, and Age plus Gender plus ALC plus LDH plus CRP in 3 independent cohorts; (A) cohort 1, (B) cohort 2, and (C) cohort 3.

**Figure S4.** Longitudinal NfL, ALC, CRP, and LDH levels in critical COVID‐19 patients (survived versus died), plotted with respect to the number of days since hospital admission.

**Figure S5** In cohort 3, patients were divided into age‐based subgroups and then plasma NfL levels were compared across survived versus died patients using Mann–Whitney *t*‐test.

**Data S1.** Cohort, demographics, disease, and severity diagnosis, timeline of important events during disease, timeline of sample analysis, NfL – raw and HC age‐adjusted measurements, comorbidities, and lab test measurements for systemic markers (ALC, CRP, and LDH) data for all subjects (HC = 76, non‐COVID‐19 acute = 10, MS non‐active = 35, MS active = 35, COVID‐19: moderate = 10, critical: survived = 195, and critical: deceased = 133).

**Appendix S1.** Supplementary results.
