# Supplementary figures and images for: Prognostic value of serum/plasma neurofilament light chain for COVID‐19‐associated mortality
Source: Ann Clin Transl Neurol. 2022 Mar 21;9(5):622–32. doi: 10.1002/acn3.51542 (PMC9082006; doi:10.1002/acn3.51542)

# Model predictors

**A**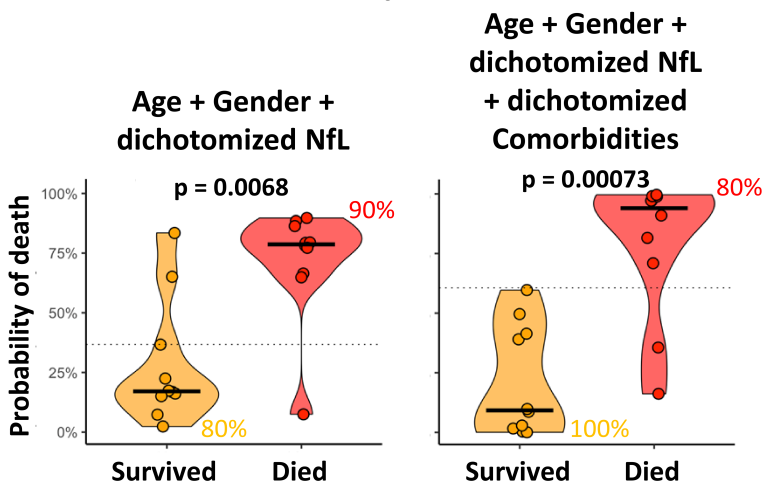**B**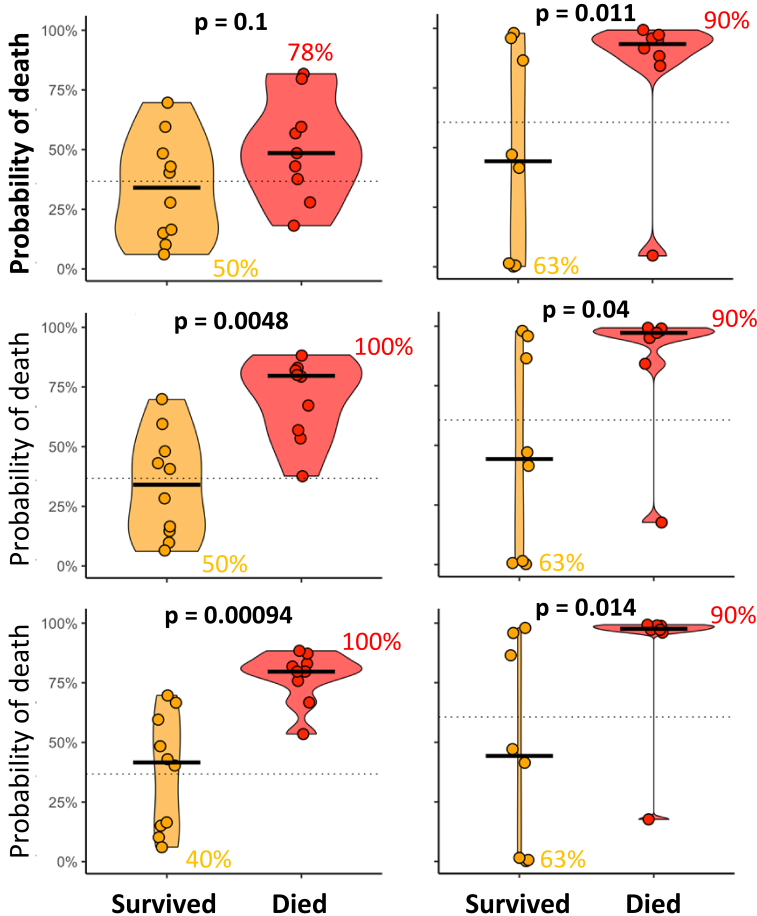**C**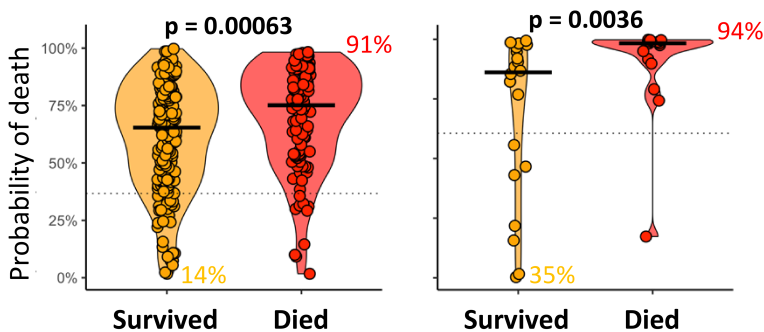

Supplement: Supplementary file 2 — Figure S2. Comparisons of two predictive models of COVID‐19 associated mortality: age plus gender plus dichotomized NfL and age plus gender plus dichotomized NfL plus dichotomized comorbidities in 3 independent cohorts; (A) cohort 1, (B) cohort 2, and (C) cohort 3. [file ACN3-9-622-s007.pdf]

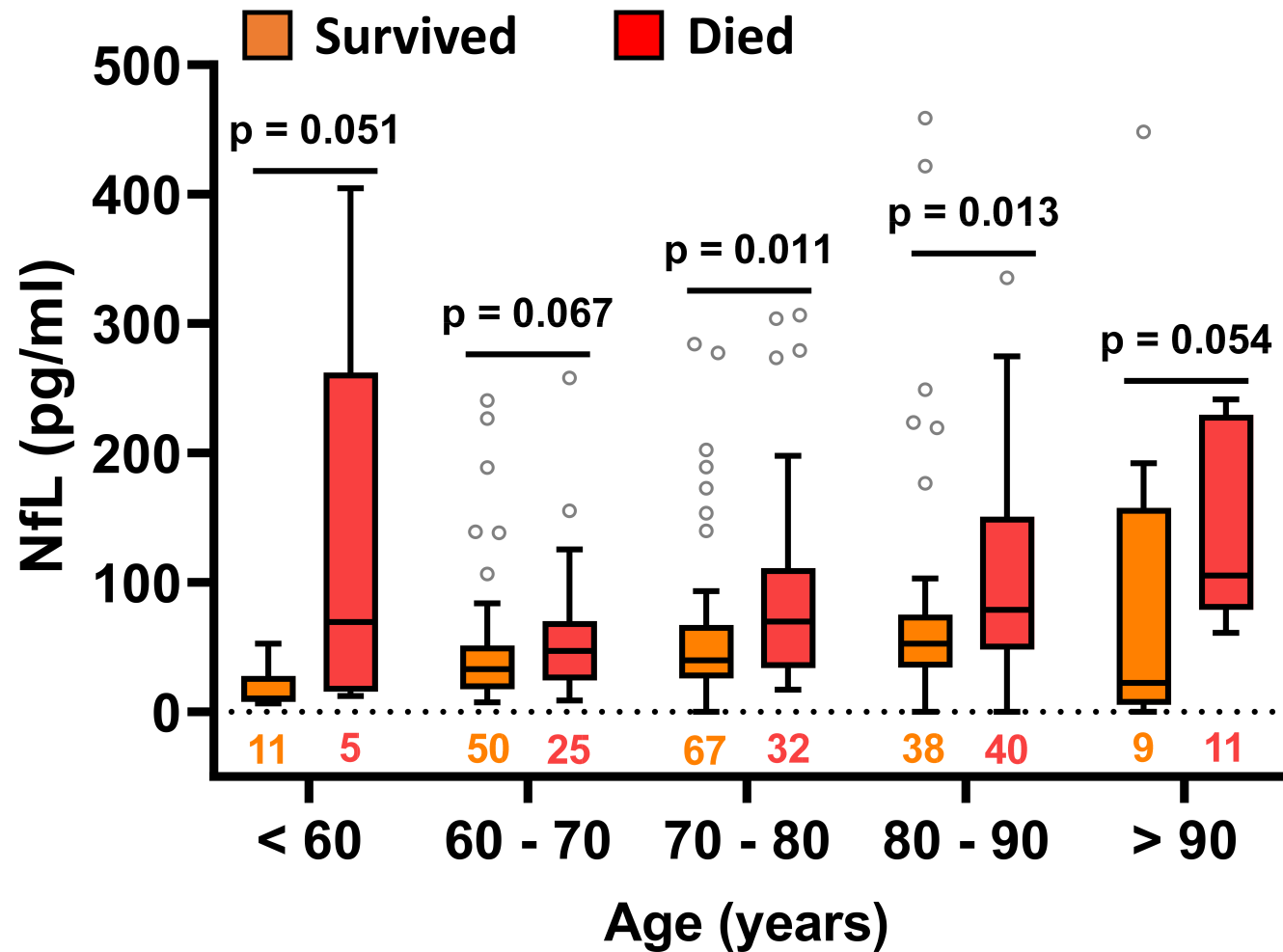

Supplement: Supplementary file 5 — Figure S5 In cohort 3, patients were divided into age‐based subgroups and then plasma NfL levels were compared across survived versus died patients using Mann–Whitney t‐test. [file ACN3-9-622-s006.pdf]
