# Supplementary material for: Prognostic value of serum/plasma neurofilament light chain for COVID‐19‐associated mortality
Source: Ann Clin Transl Neurol. 2022 Mar 21;9(5):622–32. doi: 10.1002/acn3.51542 (PMC9082006; doi:10.1002/acn3.51542)
Supplement: Supplementary file 3 — Figure S3. Comparisons of 3 predictive models of COVID‐19 associated mortality: age plus Gender, Age plus Gender plus dichotomized NfL, and Age plus Gender plus ALC plus LDH plus CRP in 3 independent cohorts; (A) cohort 1, (B) cohort 2, and (C) cohort 3. [file ACN3-9-622-s002.pdf]

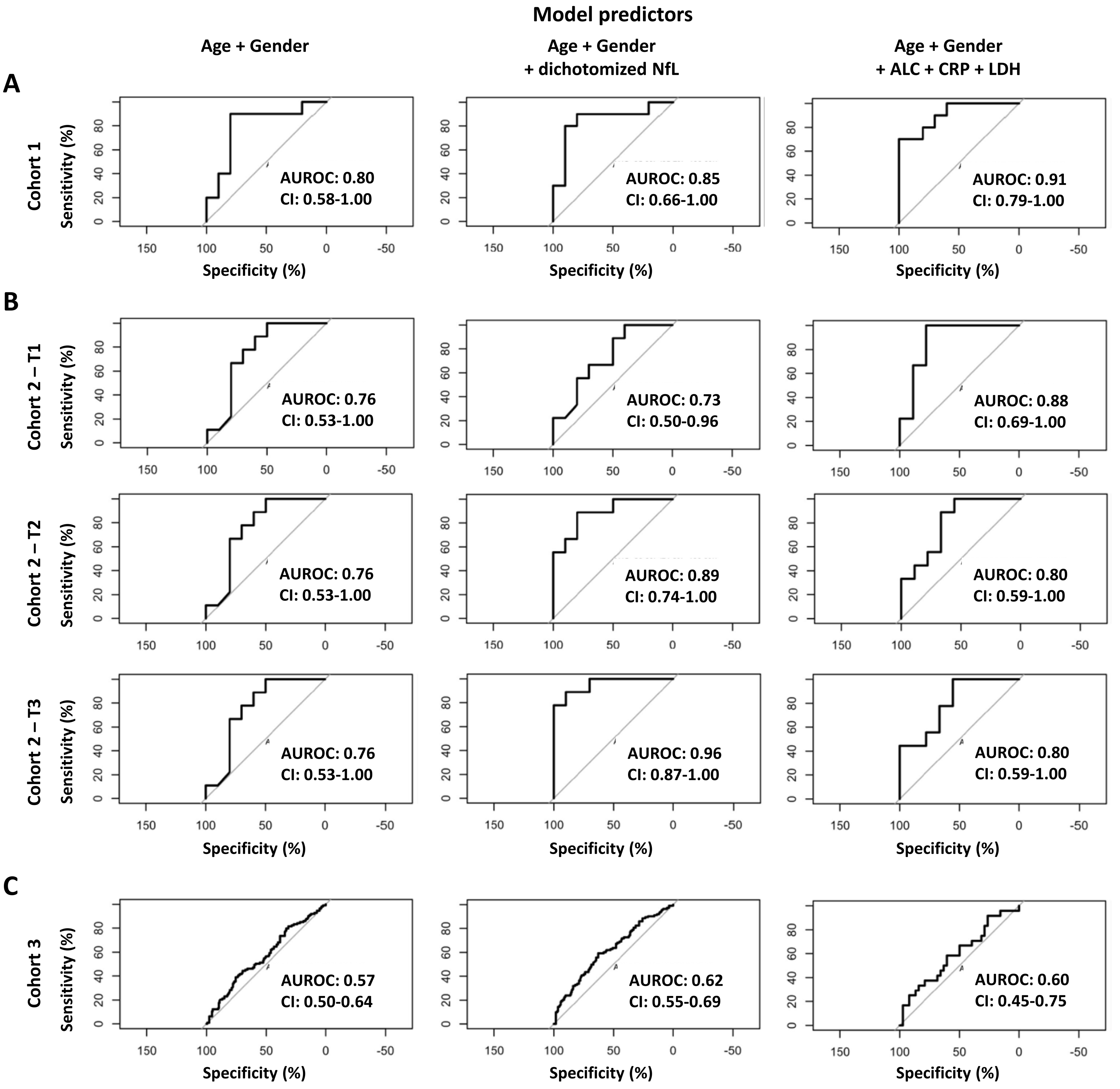
Model predictors
Age + Gender
Age + Gender + dichotomized NfL
Age + Gender + ALC + CRP + LDH
A
B
C
Cohort 1
Cohort 2 – T1
Cohort 2 – T2
Cohort 2 – T3
Cohort 3
Sensitivity (%)
Specificity (%)
AUROC: 0.80
CI: 0.58-1.00
AUROC: 0.85
CI: 0.66-1.00
AUROC: 0.91
CI: 0.79-1.00
AUROC: 0.76
CI: 0.53-1.00
AUROC: 0.73
CI: 0.50-0.96
AUROC: 0.88
CI: 0.69-1.00
AUROC: 0.76
CI: 0.53-1.00
AUROC: 0.89
CI: 0.74-1.00
AUROC: 0.80
CI: 0.59-1.00
AUROC: 0.76
CI: 0.53-1.00
AUROC: 0.96
CI: 0.87-1.00
AUROC: 0.80
CI: 0.59-1.00
AUROC: 0.57
CI: 0.50-0.64
AUROC: 0.62
CI: 0.55-0.69
AUROC: 0.60
CI: 0.45-0.75
