# Supplementary material for: Prognostic value of serum/plasma neurofilament light chain for COVID‐19‐associated mortality
Source: Ann Clin Transl Neurol. 2022 Mar 21;9(5):622–32. doi: 10.1002/acn3.51542 (PMC9082006; doi:10.1002/acn3.51542)
Supplement: Supplementary file 4 — Figure S4. Longitudinal NfL, ALC, CRP, and LDH levels in critical COVID‐19 patients (survived versus died), plotted with respect to the number of days since hospital admission. [file ACN3-9-622-s003.pdf]

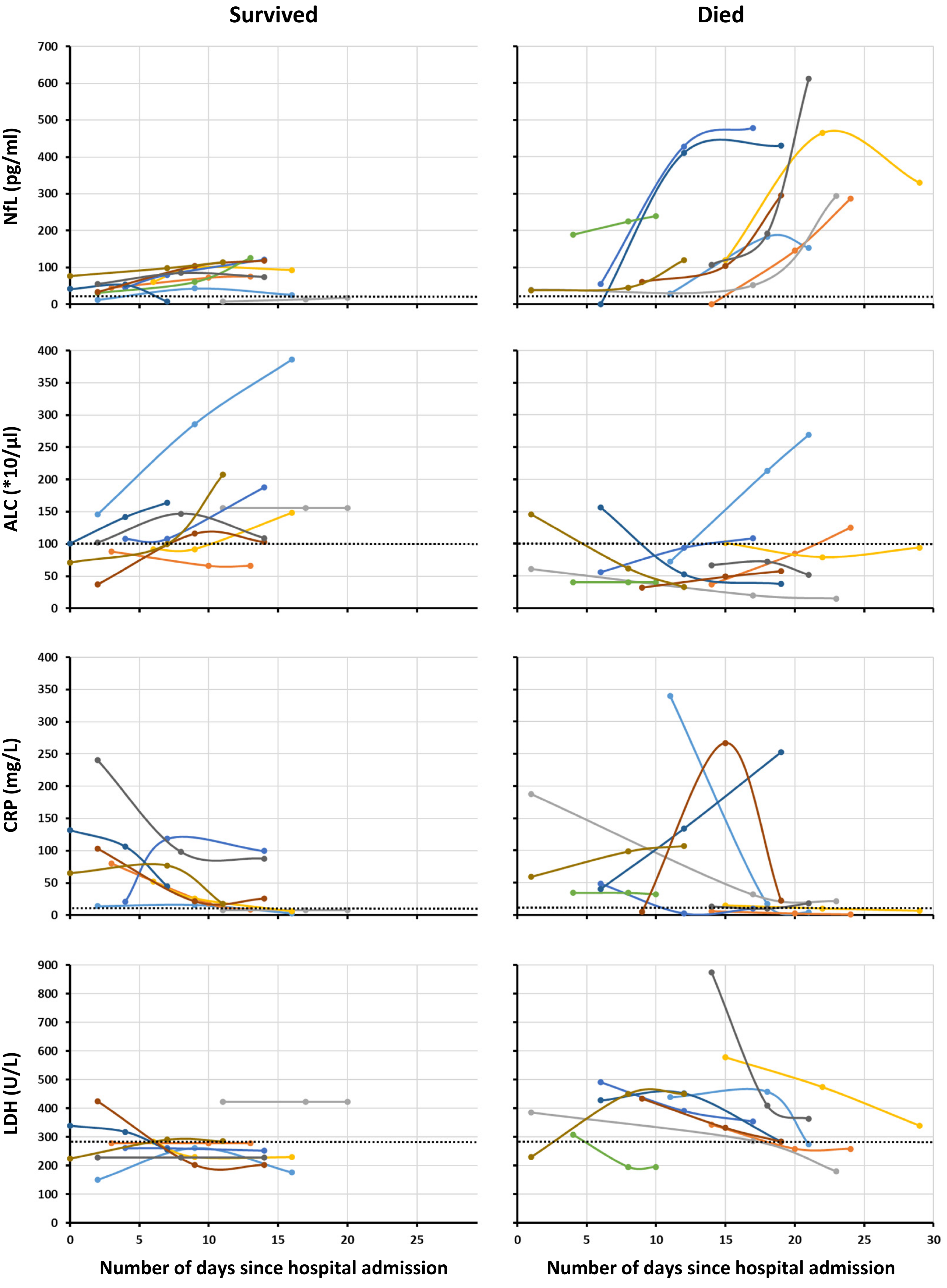
Survived
Died
NfL (pg/ml)
ALC (*10/µl)
CRP (mg/L)
LDH (U/L)
Number of days since hospital admission
Number of days since hospital admission
