# Supplementary material for: Prognostic value of serum/plasma neurofilament light chain for COVID‐19‐associated mortality
Source: Ann Clin Transl Neurol. 2022 Mar 21;9(5):622–32. doi: 10.1002/acn3.51542 (PMC9082006; doi:10.1002/acn3.51542)
Supplement: Supplementary file 7 — Appendix S1. Supplementary results. [file ACN3-9-622-s001.docx]

**Supplementary Results**

***Representation of cohort 2 data against days since hospital admission***

When plotting measurements from longitudinal cohort 2 against number of days since hospital admission (Supplementary Figure 4), we noticed change in biomarker levels (rise in NfL and LDH and decrease in ALC) only in patients those who eventually succumbed to death.

***Division of cohort 3 into age-based subgroups***

For better understanding of NfL as a biomarker of COVID-19 associated mortality across different age groups, in cohort 3, all patients were divided into age-based subgroups (<60, 60 -70, 70 -80, 80 – 90 and > 90 years; Supplementary Figure 5) and then plasma NfL levels were compared across survived versus died subgroups. In all age subgroups, NfL showed a trend of elevated levels in died patients compared those who survived but reached the statistical significance (p < 0.05) only in two age subgroups: 70 – 80 and 80 – 90 years.
