# Supplementary material for: Prognostic value of serum/plasma neurofilament light chain for COVID‐19‐associated mortality
Source: Ann Clin Transl Neurol. 2022 Mar 21;9(5):622–32. doi: 10.1002/acn3.51542 (PMC9082006; doi:10.1002/acn3.51542)
Supplement: Supplementary file 1 — Figure S1. In cohort 2, longitudinal plasma NfL (blue), ALC (orange), CRP (yellow), and LDH (green) levels in critically ill COVID‐19 patients those who survived, plotted with respect to the number of days before discharge. [file ACN3-9-622-s005.pdf]

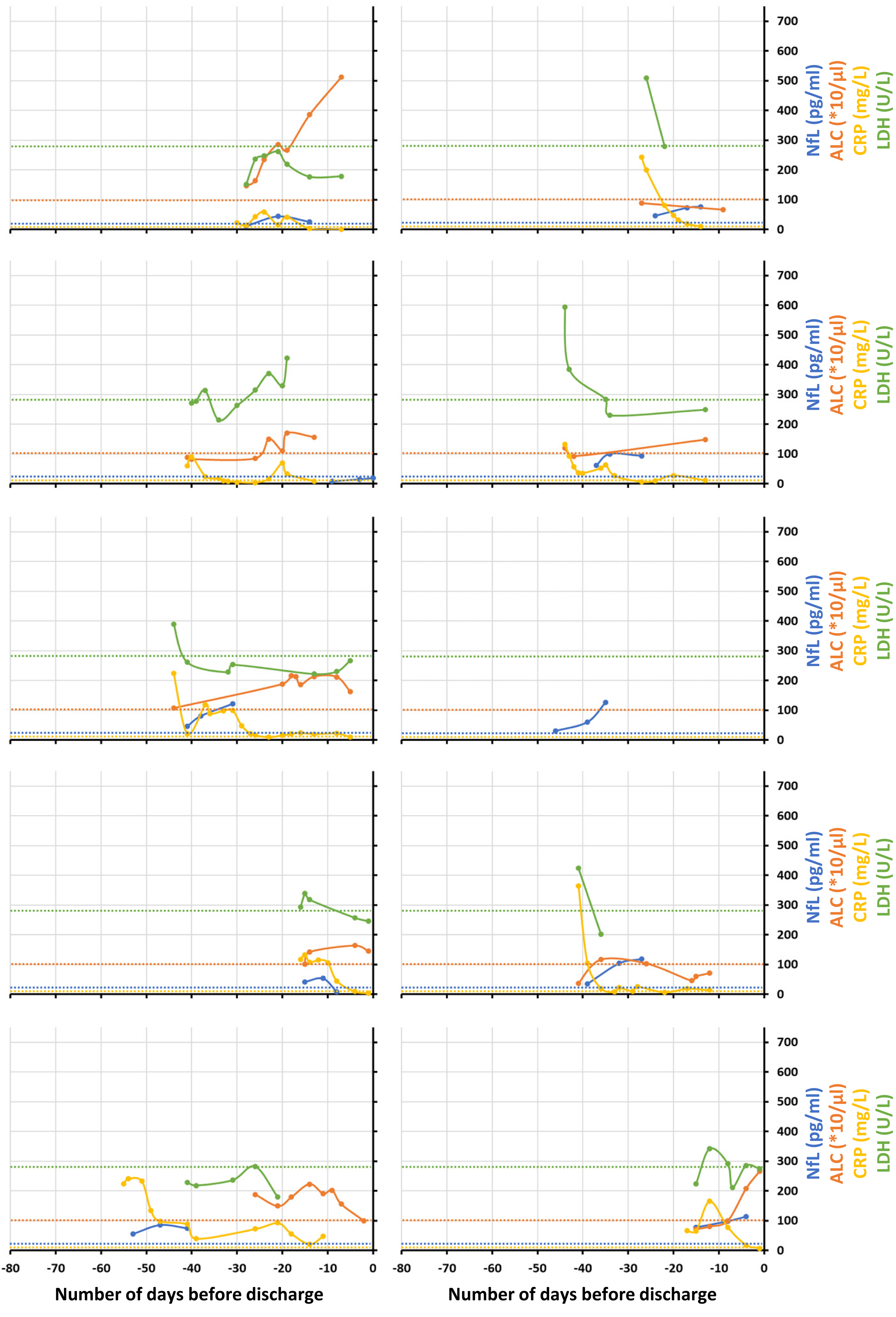

NfL (pg/ml)
ALC (*10/µl)
CRP (mg/L)
LDH (U/L)
Number of days before discharge
Number of days before discharge
